# The role of auditory transient and deviance processing in distraction of task performance: a combined behavioral and event-related brain potential study

**DOI:** 10.3389/fnhum.2013.00352

**Published:** 2013-07-11

**Authors:** Stefan Berti

**Affiliations:** Department for Psychology, Johannes Gutenberg-University MainzMainz, Germany

**Keywords:** auditory distraction, control of attention, event-related brain potentials, mismatch negativity (MMN), P3a, reorienting negativity (RON), predictive coding

## Abstract

Distraction of goal-oriented performance by a sudden change in the auditory environment is an everyday life experience. Different types of changes can be distracting, including a sudden onset of a transient sound and a slight deviation of otherwise regular auditory background stimulation. With regard to deviance detection, it is assumed that slight changes in a continuous sequence of auditory stimuli are detected by a predictive coding mechanisms and it has been demonstrated that this mechanism is capable of distracting ongoing task performance. In contrast, it is open whether transient detection—which does not rely on predictive coding mechanisms—can trigger behavioral distraction, too. In the present study, the effect of rare auditory changes on visual task performance is tested in an auditory-visual cross-modal distraction paradigm. The rare changes are either embedded within a continuous standard stimulation (triggering deviance detection) or are presented within an otherwise silent situation (triggering transient detection). In the event-related brain potentials, deviants elicited the mismatch negativity (MMN) while transients elicited an enhanced N1 component, mirroring pre-attentive change detection in both conditions but on the basis of different neuro-cognitive processes. These sensory components are followed by attention related ERP components including the P3a and the reorienting negativity (RON). This demonstrates that both types of changes trigger switches of attention. Finally, distraction of task performance is observable, too, but the impact of deviants is higher compared to transients. These findings suggest different routes of distraction allowing for the automatic processing of a wide range of potentially relevant changes in the environment as a pre-requisite for adaptive behavior.

## Introduction

The detection of unexpected changes in the sensory environment is a central prerequisite for flexible adaptation to new situations in a dynamic environment: Automatic processing of currently irrelevant sensory input can result in distraction of ongoing behavior, allowing for an evaluation of changes in the environment. In other words, salient changes in the environment may automatically trigger orientation of attention. This is very prominent in the auditory modality and enables scanning of the surrounding environment without physical orientation to sound locations. With this, it is possible to detect sudden onsets or changes irrespective of attentional allocation to the sound source. Importantly, the pre-attentive detection of changes in the auditory environment covers different types of changes spanning from sudden onsets of a sound (e.g., a ring-tone of a mobile phone during a lecture) to small variations within a continuous sound stream (e.g., prosodic changes in the lecturer's speech in response to the ring-tone). Consequently, it was argued that different neuro-cognitive mechanisms exist for tapping the broad range of potentially relevant changes in the environment (Näätänen, 1990; Escera et al., 1998; Rinne et al., 2006; Näätänen et al., 2007; Winkler et al., 2009; Berti, 2012). The present study aims at comparing these two different situations of auditory change detection directly and at testing whether rare auditory stimuli may trigger different mechanisms of sensory processing and subsequent attentional orientation depending on whether they either are deviating from a continuous stimulation or are transient auditory events.

In recent years a number of studies have dealt with the processing of auditory stimuli or stimulus features which were irrelevant for the current task. Under special circumstances, a rare change of a task irrelevant part of the auditory stimulation results in behavioral distraction, i.e., it is mirrored in prolonged response times and a decreased accuracy in processing the experimental task (for a review see Escera et al., 2000). In these studies the logic of the oddball paradigm is applied, which is that two types of stimuli are presented with one frequent stimulus (e.g., in 87% of the trials; the so called standard) and one rare stimulus (e.g., in 13% of the trials; the so called deviant). Standard and deviant stimuli can, for instance, differ in pitch (e.g., 1000 vs. 1100 Hz). However, in distraction paradigms this variation of standard and deviant pitch is task irrelevant and can be ignored because the participants' task is related to another stimulus feature. For instance, in an auditory-auditory distraction paradigm introduced by Schröger and Wolff (1998), the presented auditory stimuli differed in duration (half of the stimuli had a duration of 200 ms and the other half of 400 ms) and the participants were instructed to perform a duration discrimination task. Tone duration and pitch varied independently from each other and the presentation of the deviant pitch could not be anticipated. In an auditory-visual cross-modal distraction paradigm (see Escera et al., 1998) task relevant visual stimuli were preceded by task irrelevant auditory standard or deviant tones. In addition to these two types of distraction paradigm, visual-visual (e.g., Berti and Schröger, 2004), bimodal (Boll and Berti, 2009), and recently vibrotactile-visual (Parmentier et al., 2011b; Ljungberg and Parmentier, 2012) paradigms were developed which resemble the general distraction paradigm logic. The intriguing finding is that irrelevant stimulus features in all these different types of paradigms distract task performance, suggesting that the processing of deviants is highly important. It has been argued that distraction by deviants mirrors the openness for changes in the environment in order to enable fast switches of attention to a potentially relevant stimulus (see, for instance, Escera et al., 2000; Berti and Schröger, 2003; Berti, 2008a). This assumption was investigated by Hölig and Berti (2010) by combining the distraction logic with the task-switching logic, demonstrating that deviants indeed allow for a fast task switch and do not disrupt information processing in general. This mirrors the idea of an orienting response (OR, see Sokolov, 1963, 1990; Barry, 2009) as a basic mechanism of adaptation to changes in the environment. Finally, recent behavioral studies indicate that the automatic orientation of attention toward new information triggers an involuntary semantic evaluation of it (Parmentier, 2008; Parmentier et al., 2011c, 2013) which further supports the interpretation of distraction as a relevant adaptive mechanism.

On a functional level, it has been argued that three processing steps in the neuro-cognitive system are underlying behavioral distraction (see, for instance, (Escera et al., 2000; Berti, 2008a; Horváth et al., 2008), namely (1) pre-attentive change detection, (2) involuntary orienting of attention, and (3) voluntary reorienting of attention. In more detail, the initial step of change detection is assumed to automatically process the incoming sensory information and to detect novel or deviating aspects in the physical input indicating changes in the environment. In case of the detection of a change the second step is triggered, which is the orientation of attention onto the new information; this orientation of attention is assumed also to proceed automatically and constitutes involuntary allocation of attention. However, in case the change in the environment is irrelevant (as is the case in the above mentioned distraction paradigms) a reorientation of attention to the relevant aspects of the sensory input is necessary in order to perform the task at hand. Therefore, the third processing step involved in distraction is voluntary reorientation of attention. Taken together, these additional steps of involuntary and voluntary switching of attention triggered by change detection disturb the processing of the task relevant information, resulting in prolonged responses and/or increased error rates (see, for instance, Schröger, 1996; Alho et al., 1997; Escera et al., 1998). This sequence of distraction related neuro-cognitive processes is mirrored in the human event-related brain potential (ERP). In detail, in distraction paradigms focusing on processing of (irrelevant) auditory stimuli (as is the case in the auditory-auditory and auditory-visual paradigm) a typical sequence of components is observable in the deviant compared to the standard ERP, mirroring sensory processing and the control of attention related with the behavioral distraction effects (see, for instance, Schröger and Wolff, 1998; Escera et al., 2001; Berti et al., 2004; Berti, 2008a, 2012; Horváth et al., 2008; Hölig and Berti, 2010; for a review see Escera and Corral, 2007): Task irrelevant auditory changes elicit the mismatch negativity (MMN), P3a, and reorienting negativity (RON), with the MMN indexing the pre-attentive sensory processing (Näätänen, 1990), the P3a indexing the involuntary switch of attention onto the new information (Friedman et al., 2001), and the RON indexing the subsequent reorienting back to the task relevant information (Schröger and Wolff, 1998; Berti, 2008a). However, for several reasons this “mechanistic” view of a three step processing chain underlying distraction seems to be too simple for tapping the functional diversity of flexible adaptation to ongoing changes in the sensory environment.

The analysis of behavioral data in a number of studies demonstrated a variety of factors influencing the actual effect of irrelevant deviating sensory information on task performance, including different types of top-down effects (Berti and Schröger, 2003; Sussman et al., 2003; Munka and Berti, 2006; Wetzel and Schröger, 2007; SanMiguel et al., 2008; Ruhnau et al., 2010; Horváth and Bendixen, 2012; Parmentier and Hebrero, 2013), the degree of the change compared with the standard stimulation (Yago et al., 2001; Berti et al., 2004; Berti, 2012), or the local micro-structure of the stimulation, i.e., with regard to the pattern of standard repetitions and change (Bendixen et al., 2007; Jankowiak and Berti, 2007; Horváth et al., 2008). In addition, Parmentier et al. (2010) and Wetzel et al. (2012) also reported potential facilitation effects by deviant stimuli, raising the question why and when auditory deviants become distracting (see also Parmentier, 2008; Parmentier et al., 2011a). One potential answer to this question is that pre-attentive change detection and subsequent triggering of attentional allocation can be based on different mechanisms optimized for different potentially relevant events in the auditory environment (Näätänen, 1990; Escera et al., 1998; Rinne et al., 2006; Näätänen et al., 2007; Horváth et al., 2008; Winkler et al., 2009; Berti, 2012). For instance, Escera et al. (1998) presented two types of auditory changes within the auditory-visual distraction paradigm: a deviant sinusoidal tone with a frequency of 700 Hz and novel stimuli which comprised short environmental sounds and which were presented only once within the experiment; the standard stimuli (sinusoidal tones with a pitch of 600 Hz) were presented in 80% of the trials while deviant and novel stimuli were presented in 10% of the trials each. In this study, deviants and novels both distracted visual task performance but distraction effects on the behavioral and ERP levels showed interesting differences: On the one hand, deviants resulted in a stronger distraction effect than novels. This is a surprising result because novels comprise a stronger change compared with deviants. On the other hand, novels elicited an increased N1 component while deviants elicited an MMN in the ERP. Moreover, both, deviants and novels elicited a subsequent P3a but the P3a in novel trials comprised of two subcomponents with an early and a later subcomponent. The differential effects of novels and deviants on the early, deviance related ERP components were replicated in a study by Berti (2012) applying the auditory-visual distraction paradigm. In this study, three types of rare (13% of the trials in each block) auditory changes were presented before the relevant visual stimulus: a pitch deviant (pitch increase of 10%), novel stimuli, and—in addition to the Escera et al. (1998) study—a short environmental sound. In detail, the short environmental sound was a sound that is similar to the novel stimuli and, therefore, differs in sensory richness compared with the sinusoidal standard stimulus. But in contrast with the novel stimuli this sound is repeated as a rare stimulus and, therefore, is not novel within the experiment. The ERPs showed MMN elicited by the deviants, but N1 increase plus MMN with the two other types of changes (environmental sound or novels). Interestingly, behavioral distraction effects were obtained in the conditions with the strong changes (i.e., the novels and the environmental sound) only. Finally, in a study by Rinne et al. (2006) applying the auditory-auditory distraction paradigm, intensity decrement deviants elicited an MMN only while intensity increment deviants elicited an MMN preceded by an N1 enhancement. Taken together, this pattern of results suggests two different mechanism of change detection: One mechanism capable of detecting salient changes like the onset of a pronounced difference, a novel or a transient sound in the environment which is mirrored in the N1 component, and another mechanism capable of detecting slight or small changes in the sensory input (see Escera et al., 1998; Rinne et al., 2006; Berti, 2012). According to Näätänen (1990) the first mechanism can be denoted as transient detector and the second mechanism as deviance detector (see also Näätänen et al., 2007; Winkler et al., 2009).

With regard to the deviance detection mechanism, it was assumed that the MMN mirrors the processing of the violation of the current sensory input from a sensory memory trace built on basis of the ongoing (standard) stimulation (see, for instance, Näätänen, 1990; Schröger, 1997; Näätänen et al., 2005). Recently, this “memory based” interpretation of the MMN was developed into a more general interpretation within the context of predictive coding theory (see, for instance, Winkler, 2007; Winkler and Czigler, 2012). In this framework, the MMN is assumed to reflect the “prediction error” which is the difference between the expected sensory input (as predicted from the previous input) and the actual sensory input. A basic prediction might be that the ongoing stimulation will be continued by a physically identical stimulus, i.e., the standard (this resembles the memory based MMN), but predictions about the upcoming stimulation might be derived on the basis of more complex rules or regularities (see Bendixen et al., 2007; for a review see Näätänen et al., 2001). However, this interpretation suggests that deviance processing as a basis for distraction relies on the existence of additional information allowing for building up a memory trace or deriving predictions regarding the sensory environment. In contrast, it remains unclear whether also rare changes not embedded within a continuous stream of sensory information are capable of triggering change detection and switching of attention resulting in distraction of ongoing task performance. This question is addressed in the present study by applying the auditory-visual distraction paradigm (see Escera et al., 1998). In detail, rare auditory stimuli are presented in two conditions. In one condition, these rare stimuli are embedded within a stream of frequent stimuli (i.e., standard stimuli; Oddball condition) while in the other condition, the same type of stimuli are presented infrequently before the task relevant visual stimulus but there is no second type of stimulus which could serve as a standard stimulation (Distractor condition). On the one hand, rare stimuli in the Oddball condition constitute typical deviant stimuli and should, therefore, result in the elicitation of deviance related behavioral and ERP distraction effects including the elicitation of an MMN. On the other hand, rare stimuli in the Distractor condition should elicit a pronounced N1 (see Näätänen and Picton, 1987). The question is whether this N1-based transient detection will also trigger a behavioral distraction effect and attention related ERP components (i.e., P3a and RON).

## Experiment 1

### Methods

#### Participants

Twelve healthy volunteers (age-span 22–38 years, mean age 28.4 years, 2 males) with normal or corrected to normal visual acuity participated in the study. In accordance with the Declaration of Helsinki all subjects gave written consent after the nature of the experiment was explained to them.

#### Experimental task

The subjects' task was to decide whether a visually presented number was odd or even by pressing one of two assigned response buttons. The visual stimuli were numbers between one and eight with a presentation time of 200 ms and a stimulus-onset asynchrony (SOA) of 1200 ms. The probability of odd and even numbers was 50% each. Subjects performed this task in two conditions: In the Oddball condition every number was preceded by a 200 ms sinusoidal tone (including 5 ms rise and fall time) with a SOA of 300 ms. Importantly, the auditory stimuli could be of a standard (600 Hz, 87% of the trials; standard trial) or a deviant (660 Hz, 13% of the trials; deviant trial) frequency. In the Distractor condition a preceding tone (660 Hz) was presented only in 13% of the trials (distractor trial) while in the rest of the trials the visual stimulus was not preceded by an auditory stimulus (no-tone trial). With regard to the frequency of the different trial types in the two conditions, standard and no-tone trials will be referred to as *frequent trials* and deviant and distractor trials as *rare trials*. The tones were presented binaurally with a sound pressure level of 75 dB. In both conditions the auditory stimuli were irrelevant for the odd-even discrimination task. The trials were presented blockwise for the Oddball and the Distractor condition. Each condition consisted of 7 blocks of 100 randomized trials, with the exception that each infrequent trial was followed by at least three frequent trials. Every block started with a fixation cross presented for 1000 ms at the middle of the screen at the position at which the visual stimuli were presented. Moreover, the experiment started with a training block consisting of 100 no-tone trials in order to practice the odd-even discrimination task. The subjects were instructed to react with a left or a right key press as fast and as accurate as possible and to reduce eye movements, blinks, and movements in general. The response-to-key mapping and the order of conditions were randomized between subjects.

#### Behavioral data analysis

Mean reaction time (RT) and hit rate were computed for the responses. Trials with RTs shorter than 200 ms were discarded as false reactions, and mean RTs were only computed for correct trials. The first frequent trial after a rare trial was excluded from the analysis. The behavioral data was subjected to Two-Way repeated-measures analysis of variance (ANOVA) with factors Condition (Oddball vs. Distractor) and Trial type (frequent trial vs. rare trial). In addition, distraction effects in RTs were calculated for each condition separately by subtracting the RT in frequent trials from the RT in rare trials and were tested for a significant difference from zero by two one-sample, two-tailed *t*-tests.

#### EEG recording and analysis

The electroencephalogram (EEG) was recorded with a 32-channel Neuroscan SynAmps amplifier from 19 electrodes placed on a cap according to the International 10–20 System (Jasper, 1958) and from two additional electrodes placed at the left (LM) and right mastoids (RM). The reference electrode was placed at the tip of the nose. The EEG was online filtered with a 0.05–70 Hz bandpass and a 50 Hz notch filter. Electro-occulograms (EOG) were also recorded for offline artifact correction vertically from above and below the right eye and horizontally from the outer canthi of both eyes. The EEG was offline bandpass filtered with a 1–30 Hz bandpass filter. The ERPs were computed separately for frequent and rare trials in the Oddball and Distractor condition within a time window from −200 to −800 ms relative to auditory stimulus onset in case of trials consisting of an auditory stimulus or the comparable point in time in the no-tone trial. In other words, ERPs were calculated relative to a point in time 300 ms before the onset of the task relevant visual stimuli in all trials. All epochs with extensive eye movements (i.e., whenever the standard deviation within a sliding 200 ms time window exceeded 25 μ V at the EOG or at Fz) were rejected automatically from the calculation of ERPs. The 200 ms period before stimulus onset or relative to the comparable point in time in the no-tone trial served as baseline. Again, the first frequent trial after a rare trial was excluded from ERP computation. In addition, difference waves were computed separately for the two conditions by subtracting the ERPs elicited by frequent trials from the ERPs elicited by rare trials. The ERPs and difference waves are depicted at Fz, Cz, Pz, and LM because these electrodes represent the pattern of results best.

After artifact correction, the data sets of two participants were excluded from further ERP analysis because of a too low number of artifact free epochs in one or more trial types (i.e., less than 40 artifact free EEG epochs). After visual inspection, the ERPs of the remaining ten participants were analyzed at Fz and Cz by calculating the mean amplitude in five different time windows (70–150, 150–220, 220–390, 390–530, and 530–790 ms) separately for the two stimulus types in the two conditions. In addition, differences between rare and frequent trial ERP amplitudes were calculated in order to test for significant change-related components by means of one-sample, two-tailed *t*-tests against zero (see Table 1). In addition, the mean amplitudes in the five respective time windows were evaluated for significant effects of the factors Trial type, Condition, and Electrode (Fz vs. Cz) by Three-Way repeated-measure ANOVAs.

**Table 1:** **Mean amplitudes (and standard error of mean) and *t*-statistics of deviance related differences between rare and frequent trial separately for the five time windows at Fz and Cz in Experiment 1**.

	**Oddball condition**				**Distractor condition**			
	**Fz**		**Cz**		**Fz**		**Cz**	
**Window (ms)**	μ **V**	***t***	μ **V**	***t***	μ **V**	***t***	μ **V**	***t***
70–150	−0.89 (0.31)	2.87^*^	−0.24 (0.41)	0.57	−7.74 (1.08)	7.91^***^	−8.42 (1.16)	7.23^***^
150–220	−2.52 (0.69)	−3.64^**^	−1.48 (0.66)	2.25^◦^	5.97 (1.32)	4.53^**^	9.56 (1.94)	4.96^***^
220–390	1.91 (0.51)	3.72^**^	1.87 (0.50)	3.75^**^	3.22 (0.48)	6.76^***^	3.56 (0.39)	9.04^***^
390–530	−0.28 (0.34)	0.82	0.25 (0.38)	0.67	−2.53 (0.44)	5.81^***^	−1.95 (0.43)	4.54^**^
530–790	−0.90 (0.24)	3.70^**^	−0.68 (0.25)	2.67^*^	−1.00 (0.19)	5.21^***^	−1.31 (0.25)	5.25^***^

All df's = 9; significance levels:

◦ p < 0.1,

* p < 0.05,

** p < 0.01,

*** p < 0.001.

### Results

**Figure 1:**
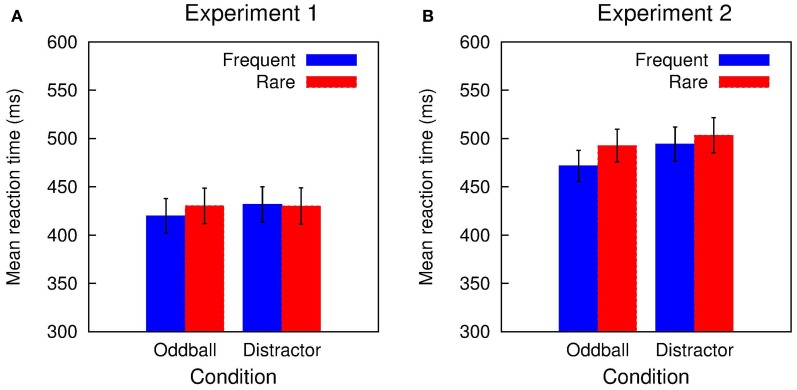
**Summary of the RT results obtained in (A) Experiment 1 and (B) Experiment 2**. A small distraction effect is elicited in rare stimuli in the Distractor condition of Experiment 1. In Experiment 2, rare stimuli in both conditions resulted in RT prolongation.

In Experiment 1, accuracy in the visual classification task was high (range of hit rate: 0.90-0.91) and did not differ between the two conditions and trial types (all *F*'s in the Condition × Trial type ANOVA < 1). Figure 1A depicts the mean RTs obtained in Experiment 1: RTs were in the range of 420 to 432 ms and showed only small variations between the two conditions and trial types. The Two-Way ANOVA revealed only a marginal significant interaction of Condition and Trial type while neither the Condition nor the Trial type obtained a significant effect: Condition *F*_(1, 11)_ < 1, *p* = 0.410, η^2^_*p*_ = 0.063; Trial type *F*_(1, 11)_ = 1.424, *p* = 0.258, η^2^_*p*_ = 0.115; Condition × Trial type, *F*_(1, 11)_ = 3.391, *p* = 0.093, η^2^_*p*_ = 0.236. This was also mirrored in the distraction effects (rare trial RT minus frequent trial RT; DRT) which was small in the Oddball condition and close to zero in the Distractor condition: Oddball DRT 10 ms, *t*_(11)_ = 1.924, *p* = 0.081, Cohen's *d* = 0.163; Distractor DRT −2 ms, *t*_(11)_ < 1.

**Figure 2:**
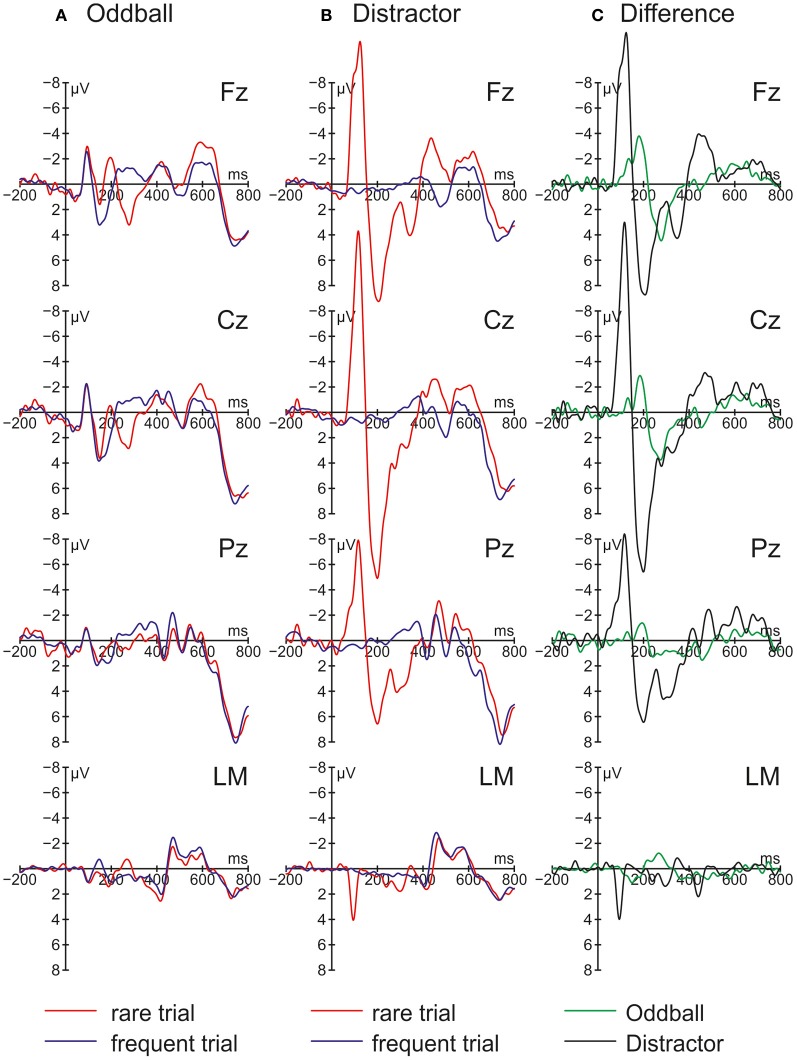
**Grand average event-related brain potentials (ERPs) and difference waves obtained in Experiment 1 (*N* = 10)**. ERPs are depicted separately for rare and frequent trials in **(A)** the Oddball condition and **(B)** the Distractor condition. **(C)** Difference waves are computed by subtracting frequent trial ERPs from rare trial ERPs.

Figure 2 summarizes the ERPs obtained in the Oddball (Figure 2A) and Distractor condition (Figure 2B). In the Oddball condition, both types of auditory stimuli elicited the N1 component which is followed by a second negativity in the rare but not in the frequent stimulus type (see Figure 2A, Fz). In contrast, the rare auditory stimulus in the Distractor condition elicited a distinctive N1 followed by a positive peak around 200 ms (see Figure 2B, Fz). The difference between rare and frequent tones in the two conditions is depicted in the difference waves (Figure 2C). The difference waves illustrate that the early negative components elicited by rare auditory stimuli in the Oddball and the Distractor condition differ with regard to their peak latencies. In other words, the processing of deviant tones in the Oddball condition are mirrored in the MMN while the processing of distractor tones in the Distractor condition are mirrored in the N1. In addition, both the N1 and the MMN are followed by positive components: In the Oddball condition a P3a peaking around 280 ms is visible. In the Distractor condition two positive peaks are visible at Fz: a P2 peaking around 200 ms and a P3a peaking around 340 ms. Finally, an early phase of the RON component is obtained in the Distractor condition between 400 and 520 ms and a later phase of the RON component is obtained in both conditions between 520 and 700 ms. The statistical tests of the mean amplitudes in the difference waves (by means of *t*-tests against zero) confirm the elicitation of N1/MMN, P3a, and late RON in the Oddball condition and the elicitation of N1, P2, P3a, and early and late RON in the Distractor condition (see Table 1). The results of the statistical analysis for the five time windows by means of 2 × 2 × 2 repeated-measure ANOVA is summarized in Table 2: Main effects of the factor Stimulus type are obtained in all time windows while main effects of the factor Condition are confined to the three early time windows. In addition, significant interaction terms in all except the latest time windows confirm the differences in processing of rare auditory stimuli in the two conditions. This difference is already mirrored in the comparison of the ERPs of the rare stimuli: In all but the latest time window, deviant ERP amplitudes differ from distractor ERP amplitudes at FZ: 70–150 ms: *t*_(9)_ = 7.27, *p* < 0.0001, Cohen's *d* = 2.39; 150–220 ms: *t*_(9)_ = 3.81, *p* = 0.0042, Cohen's *d* = 2.03; 220–390 ms: *t*_(9)_ = 6.13, *p* = 0.0002, Cohen's *d* = 1.39; 390–530 ms: *t*_(9)_ = 2.52, *p* = 0.0328, Cohen's *d* = 0.85; 530–790 ms *t*_(9)_ < 1.

**Table 2:** **Statistical evaluation of effects of Trial type (rare vs. frequent), Condition (Oddball vs. Distractor), and Electrode (Fz vs. Cz) by means of repeated-measure ANOVAs separately for the five time windows in Experiment 1**.

**Time window**	**70–150**		**150–220**		**220–390**		**390–530**		**530–790**	
**Factor**	***F***	η**^2^_*p*_**	***F***	η**^2^_*p*_**	***F***	η**^2^_*p*_**	***F***	η**^2^_*p*_**	***F***	η**^2^_*p*_**
Trial type (T)	63.49^***^	0.876	22.73^**^	0.716	49.31^***^	0.845	19.43^**^	0.683	29.32^***^	0.765
Condition (C)	73.65^***^	0.891	9.299^*^	0.508	39.56^***^	0.815	<1	<0.01	<1	<0.01
Electrode (E)	<1	0.067	21.1^**^	0.701	<1	<0.01	<1	<0.01	19.55^**^	0.685
T × C	48.14^***^	0.842	21.34^**^	0.703	9.195^*^	0.505	14.28^**^	0.613	1.603	0.151
T × E	<1	<0.01	25.44^***^	0.738	<1	<0.01	14.87^**^	0.623	<1	<0.01
C × E	8.178^*^	0.476	2.953	0.246	<1	<0.01	<1	<0.01	2.095	0.189
T × C × E	2.053	0.186	8.162^*^	0.476	1.165	0.115	<1	<0.01	19.75^**^	0.687

F-values and partial η_*p*_^2^'s are summarized; all df's = 1, 9; significance levels:

* p < 0.05,

** p < 0.01,

*** p < 0.001.

### Discussion

Rare pitch changes in the Oddball condition elicited the MMN, the P3a, and the RON and result in a small (but statistically not significant) RT prolongation. This pattern of results can be best interpreted as distraction of the visual odd-even classification task by the task irrelevant change of the preceding auditory stimuli. By comparison, the infrequent presentation of the same stimuli in the Distractor condition elicited a pronounced N1 component followed by a P2, a late P3a, and the early and the late RON subcomponents (see Berti, 2008a). On the behavioral level, no distraction effects were obtained in the Distractor condition. The pattern of ERP results in the two conditions mirrors the findings by Escera et al. (1998) and partly by Berti (2012): The small change (i.e., the deviant) in the Oddball condition elicited an MMN component followed by the attention related ERP components P3a and RON while the strong change (i.e., the rare transient sound) elicited a pronounced N1 component followed by an early and a late fronto-central positive component (in Berti, 2012, there is only one P3a obtained) and a RON component (visible but not analyzed in Escera et al., 1998). With regard to the fronto-central positive components between 150 and 390 ms, these resemble the early and late P3a reported in Escera et al. (1998; see also Berti, 2008b) but in the present Experiment 1 the early positive component peaks around 200 ms (230 ms in Escera et al., 1998). With this, it is questionable whether the first positive component following the N1 is best described as a P2 or an early P3a. In contrast, the later positive peak around 340 ms with a frontal maximum resembles a typical P3a and together with the subsequent RON component one may assume that also the rare sinusoidal tones in the Distractor condition resulted in attentional switching and distraction of task performance. Therefore, the present results are in line with studies demonstrating the contribution of a transient detection mechanism to distraction (see Escera et al., 1998; Rinne et al., 2006; Berti, 2012). Moreover, in contrast to the studies by Escera et al. (1998), Rinne et al. (2006), and Berti (2012) the N1 elicited by the rare auditory stimuli is not followed by an MMN. This suggests that the N1-route of change detection is also capable of triggering a switching of attention without the possibility of comparing the actual input with a prediction or memory trace and elicitation of an MMN. On the other hand, this conclusion is weakened by an unclear pattern of results on the behavioral level: For the reason that the RT distraction effect in the Oddball condition is only marginally significant (in a two-tailed *t*-test) and the interaction term of the factors Condition and Trial type also failed to reach statistical significance on a 5% level, it is hard to tell whether the lack of a behavioral distraction effect in the Distractor condition is due to the incapability of the rare stimuli to result in distraction of task performance or whether it mirrors weak statistical power in the behavioral data only. This is relevant because if the lack of a behavioral distraction effect is a valid finding, one may conclude that behavioral distraction is triggered by the MMN or deviance detector route only. To elucidate this question, Experiment 1 was replicated with novels as rare stimuli because these stimuli obtained stronger distraction effects in recent studies (see, for instance, Berti, 2012).

## Experiment 2

### Methods

#### Participants

Sixteen healthy volunteers (age-span 22–51 years, mean age 28.8 years, 7 males) with normal or corrected to normal visual acuity participated in the study. In accordance with the Declaration of Helsinki all subjects gave written consent after the nature of the experiment were explained to them.

#### Experimental task

The experimental task with its instruction, timing, stimulus types, conditions, number of blocks, and numbers of trials in each block was the same as in Experiment 1. In contrast to Experiment 1, the rare stimulus type in the Oddball and the Distractor condition were novel stimuli (see Escera et al., 1998): Novels are short environmental sounds with a duration of 200 ms (including 10 ms rise and fall times) which were only presented once in each condition. Again, novels in the Oddball and Distractor condition were presented in 13% of the trials (rare trials) while in the remaining 87% of the trials either the 600 Hz sinusoidal tone (Oddball condition) or no auditory stimulus (Distractor condition) preceded the visual stimulus (frequent trial). The analysis of the participants' performance in Experiment 2 corresponded to the behavioral data analysis of Experiment 1.

#### EEG recording and analysis

The recording and analysis of the EEG were the same as in Experiment 1 with the following exceptions: The EEG was recorded from nine electrodes (F4, Fz, F3, Cz, Pz, O1, O2, RM, and LM) referenced to the nose plus the vertical and horizontal EOG. After artifact correction, the data sets of seven participants were excluded from further ERP analysis because of a too low number of artifact free epochs in one or more trial types (i.e., less than 40 artifact free EEG epochs). After visual inspection, the ERPs were analyzed at Fz and Cz by calculating the mean amplitude in four different time windows (100–160, 160–260, 260–420, and 420–570 ms) separately for the two stimulus types in the two conditions. In addition, differences between rare and frequent trial ERP amplitudes were calculated in order to test for significant change-related components by means of one-sample, two-tailed *t*-tests against zero (see Table 3). The averaged amplitudes in the five respective time windows were evaluated for significant effects of the factors Trial type, Condition, and Electrode by Three-Way repeated-measure ANOVAs.

**Table 3:** **Mean amplitudes (and standard error of mean) and *t*-statistics of deviance related differences between rare and frequent trial separately for the four time windows at Fz and Cz in Experiment 2**.

	**Oddball condition**				**Distractor condition**			
	**Fz**		**Cz**		**Fz**		**Cz**	
**Window (ms)**	μ **V**	***t***	μ **V**	***t***	μ **V**	***t***	μ **V**	***t***
100–160	−1.84 (0.83)	2.22^◦^	−2.12 (0.87)	2.45^*^	−4.75 (0.8)	5.96^***^	5.71 (0.64)	8.97^***^
160–260	2.09 (0.73)	2.88^*^	3.07 (0.86)	3.58^**^	6.16 (1.4)	4.41^**^	9.34 (1.89)	4.95^**^
260–420	4.76 (1.15)	4.14^**^	4.58 (1.47)	3.12^*^	2.56 (0.61)	4.17^**^	2.33 (0.82)	2.84^*^
420–570	−1.08 (0.46)	2.36^*^	−0.8 (0.61)	1.32	−0.99 (0.5)	1.99^◦^	0.24 (0.49)	<1

All df's = 8; significance levels:

◦ p < 0.1,

* p < 0.05,

** p < 0.01,

*** p < 0.001.

### Results

Performance in the visual classification task in Experiment 2 is slightly decreased compared with Experiment 1 but still quite accurate (range of hit rate: 0.86–0.88). Again, hit rate does not differ between the two conditions and trial types: Condition *F*_(1, 15)_ < 1; Trial type *F*_(1, 15)_ < 1; Condition × Trial type *F*_(1, 15)_ = 1.042, *p* = 0.324. Figure 1B summarizes the mean RTs obtained in Experiment 2 which are in the range of 472 to 503 ms. RTs tend to be shorter in the Oddball compared with the Distractor condition and are increased in rare compared with frequent trials. This is mirrored in the Two-Way ANOVA: Condition *F*_(1, 15)_ = 4.605, *p* = 0.049, η^2^_*p*_ = 0.235; Trial type *F*_(1, 15)_ = 32.301, *p* < 0.0001, η^2^_*p*_ = 0.683; Condition × Trial type, *F*_(1, 15)_ = 5.791, *p* = 0.029, η^2^_*p*_ = 0.279. In addition, in both conditions a significant distraction effect is obtained which is pronounced in the Oddball compared to the Distractor condition: Oddball DRT 21 ms, *t*_(15)_ = 5.196, *p* = 0.0001, Cohen's *d* = 3.198; Distractor DRT 9 ms, *t*_(15)_ = 2.919, *p* = 0.011, Cohen's *d* = 0.128.

**Figure 3:**
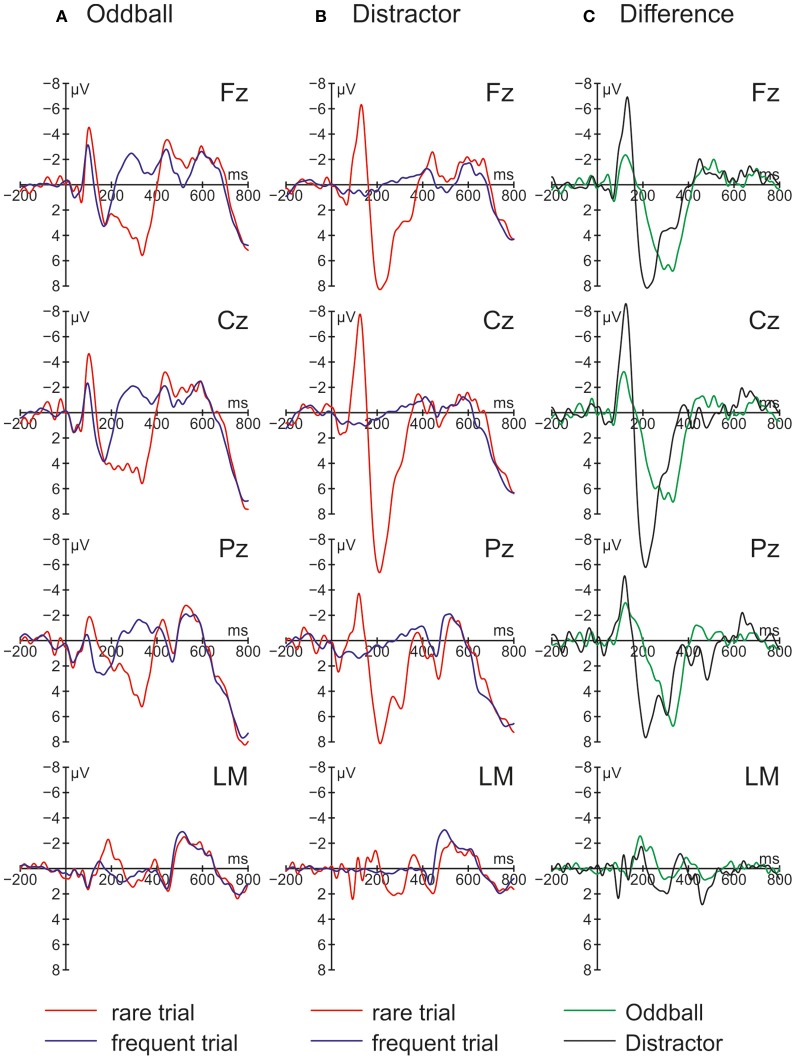
**Grand average ERPs for rare and frequent trials in (A) the Oddball condition and (B) the Distractor condition plus (C) Difference waves (i.e., rare trial ERPs minus frequent trials ERPs) obtained in Experiment 2 (*N* = 9)**.

The ERPs obtained in the Oddball and Distractor conditions are depicted in Figures 3A,B, respectively. The differences between the rare and the frequent trial type are highlighted in Figure 3C: In both conditions rare novel stimuli elicited an early negativity which is pronounced in the Distractor compared with the Oddball condition but which virtually does not differ in latency. The early negativity is followed by a positive component in both conditions but with different timings: In the Oddball condition the positivity peaks around 330 ms while in the Distractor condition it peaks around 210 ms. Subsequent to the positive peaks a late negativity is observable in both conditions at Fz between 420 and 570 ms. The existence of these fronto-central auditory change-related components is confirmed by a series of two-tailed *t*-tests (see Table 3) with one exception: In the Distractor condition the late negativity around 500 ms obtains only a marginal significant difference from zero. Table 4 summarizes the statistical analysis of the effects of the three factors Trial type, Condition, and Electrode by means of repeated-measure ANOVAs: In the early time windows, significant main effects and interactions of Trial type and Condition are revealed. In the 260–420 ms time window a significant main effect of the factor Trial type is obtained. Finally, in the latest time window a main effect of the factor Condition is revealed which is qualified by an interaction of Trial type and Electrode. Beside this interaction, the factor Electrode obtains a main effect in the 160–260 ms time window only and reveals significant interaction terms with the factor Condition in the 100–150 ms time window and with the factor Trial type and a three-way interaction in the 160–260 ms time window.

**Table 4:** **Statistical evaluation of effects of Trial type (rare vs. frequent), Condition (Oddball vs. Distractor), and Electrode (Fz vs. Cz) by means of repeated-measure ANOVAs separately for the four time windows in Experiment 2**.

**Time window**	**100–150**		**160–260**		**260–420**		**420–570**	
**Factor**	***F***	η**^2^_*p*_**	***F***	η**^2^_*p*_**	***F***	η**^2^_*p*_**	***F***	η**^2^_*p*_**
Trial type (T)	24.84^**^	0.756	20.06^**^	0.715	19.91^**^	0.713	2.637	0.248
Condition (C)	19.98^**^	0.714	8.865^*^	0.526	<1	<0.01	35.06^***^	0.914
Electrode (E)	1.09	0.119	10.32^*^	0.563	<1	<0.01	<1	<0.01
T × C	49.36^***^	0.861	25.77^**^	0.763	2.702	0.252	1.196	0.13
T × E	2.99	0.272	12.17^**^	0.603	<1	<0.01	7.013^*^	0.467
C × E	17.32^**^	0.684	4.188^◦^	0.344	<1	<0.01	1.64	0.17
T × C × E	2.754	0.256	21.12^**^	0.725	<1	<0.01	3.677^◦^	0.315

F-values and partial η^2^_p_'s are summarized; all df's = 1, 8; significance levels:

◦ p < 0.1,

* p < 0.05,

** p < 0.01,

*** p < 0.001.

### Discussion

In a nutshell, Experiment 2 replicated the findings of Experiment 1 with two major exceptions: Firstly, on a behavioral level in both conditions RT costs elicited by the novels are obtained. Importantly, the distraction effect in the Oddball condition is more pronounced than the distraction effect in the Distractor condition which is also confirmed by a Condition × Trial type interaction in the ANOVA. Secondly, novels in both conditions elicited a pronounced N1 which is more pronounced in the Distractor compared with the Oddball condition. In addition, a distinct MMN as obtained in the Oddball condition of Experiment 1 cannot be identified. The central question of Experiment 2 was whether distraction effects can be obtained in both conditions and the answer is yes. But distraction effects differ significantly with smaller RT costs in the Distractor condition, in which a prediction based processing is not possible. However, the results of Experiment 2 are best discussed together with the findings of Experiment 1.

## General discussion

Rare auditory stimuli can distract visual information processing irrespective of whether the auditory stimuli deviate from a continuous auditory stimulation or whether the auditory stimuli are transient sounds interspersed in an otherwise sound free stimulation. On the behavioral level, distraction is mirrored in RT prolongation but not in an increase of error rate. In addition, behavioral distraction effects are smaller in transient sounds compared with deviants. The ERP results suggest that distraction of task performance is triggered by two different initial processes: In transient sounds, the initial step of change detection is mirrored by the N1 and in deviants initial change detection is mirrored by the MMN; the further processing of the two types of rare stimuli share attentional processes as reflected in the P3a and RON components. These attention related ERP components are preceded by an additional positive peak around 200 ms in the transient sound processing. This suggests that distraction by rare and irrelevant sounds in different situations share later processing steps (presumably linked with controlled attention) while the initiation of distraction differs with regard to the characteristics of the context or the stimulation.

With regard to the N1 and MMN results the present findings add to the findings by Escera et al. (1998), Rinne et al. (2006), and Berti (2012) suggesting two different mechanisms of auditory change detection in the context of distraction. Here I demonstrate that the N1-route of distraction is observable when no further reference information allows for deriving predictions as a basis for change detection. On the other hand, a suddenly appearing sound within an otherwise silent environment (for example in a sound attenuated lab chamber) constitutes a strong and salient change and, therefore, does not require a reference model to be identified as a change. Interestingly, the findings in the present Distractor conditions mirror those in the study by Escera et al. (1998) where novels also constitute a strong and salient change. The present study supports the idea that the N1 reflects a transient detector mechanism (Näätänen, 1990), and from the resemblance to the findings in Escera et al. (1998) one may conclude that this transient detector also adds to the processing of the novels when these are embedded into a regular standard stimulation. This interpretation is further supported by a study by Berti (2012); however, in this study strong changes elicited an N1 enhancement irrespective of whether they were novels or not. This suggests that the triggering of the N1-route or transient detector mechanism depends on whether the stimulus is strong enough to pass a sensory threshold. Taken together, the triggering of the transient detector mechanism seems to be independent from whether the stimulus is novel or not and from whether it is embedded in a continuous stream of auditory stimulation. This is also in line with the findings by Rinne et al. (2006) reporting an additional N1 when the deviation of the standard is getting stronger (i.e., intensity increments). Moreover, the Rinne et al. (2006) study also demonstrates that in the oddball-situation the N1 might be added to the MMN-route (see also Escera et al., 1998; Berti, 2012). In other words, the two mechanisms do not exclude each other but may be processing in parallel (see Grimm and Escera, 2012, for a discussion of the variety of processes presumably underlying auditory change detection). The present study suggests that these two mechanisms can be triggered independently from each other because in Experiment 1 the pronounced N1 is confined to the Distractor condition while the MMN is confined to the Oddball condition. (But note the small N1 enhancement in Experiment 1 preceding the MMN in the Oddball condition.) The difference between the conditions is the existence of a continuous stimulation which may serve as a reference model and allow for deriving predictions about the environmental stimulation. Therefore, the findings of Escera et al. (1998), Rinne et al. (2006), and Berti (2012) also suggest that the predictive coding mechanism is not inactivated by the transient detector mechanisms and vice versa. Again, this may suggest a more parallel processing of the transient and deviant information contained in the auditory stream in the oddball stimulation. But if the transient detector and the deviant detector operate in parallel to and independently from each other, then the question arises whether and at which point in the processing chain these routes of change detection interact.

Deviants in an oddball stimulation resulting in a behavioral distraction effect usually do elicit the P3a component. This component is interpreted as an electrophysiological index of involuntary attention switching (see, for instance, Friedman et al., 2001; Escera and Corral, 2007). In the present experiments rare stimuli also elicited the P3a component. In detail, rare stimuli embedded within the standard stream showed the typical fronto-central positive peak between 300 and 400 ms irrespective of whether they are deviants or novel sounds. With regard to the (non-oddball) Distractor conditions, rare stimuli elicited a biphasic positive component with two distinct positive peaks in Experiment 1; this pattern can be interpreted as early and late P3a subcomponents (see Escera et al., 1998; Berti, 2008b). Consequently, one may conclude (1) that both the N1 and the MMN route result in the elicitation of a switch of attention and (2) that within the time window of 200 to 400 ms the effects of the two processing streams converge. The latter conclusion is in line with the findings that the P3a is elicited also by visual deviants (see Berti and Schröger, 2001, 2004) and that audio-visual interaction in bimodal deviants is visible in the P3a time window (see Boll and Berti, 2009). The first conclusion is supported by the subsequent elicitation of a RON component: This component is elicited when the detected and (at least partly) attended change is irrelevant for the task at hand with the consequence that a reorientation to the task relevant information after distraction is required (see, for instance, Schröger and Wolff, 1998; Berti et al., 2004; Berti, 2008a; Horváth et al., 2008). Experiment 1, therefore, demonstrates that N1 or transient triggered change detection is capable of eliciting involuntary and voluntary attentional allocation as demonstrated by the P3a-RON complex. This is in line with the Preliminary Process Theory (PPT; see Barry, 2009) arguing that one route contributing to OR is also based on a transient detector. Finally, this also matches the functional interpretation of distraction as a pre-requisite for a subsequent fast switch of attention or behavioral goals: As demonstrated by Berti (2008b) and Hölig and Berti (2010), switches between different objects in working memory or between different tasks are also mirrored in the P3a.

On the other hand, the present findings also challenge the interpretation of the P3a as a unitary component reflecting involuntary switching of attention for three reasons: (1) The Distractor condition obtained a pronounced early positive peak around 200 ms preceding the “classical” P3a, (2) the elicitation of a P3a is not necessarily correlated with a behavioral distraction effect (see also Munka and Berti, 2006; Wetzel et al., 2013), and (3) the degree of the change (as indexed by the N1 and MMN components) is not systematically mirrored in the P3a amplitude. The latter point is in contrast to earlier studies demonstrating a correlation of the degree of a deviation with the ERP signs of distraction, especially P3a (see Yago et al., 2001; Berti et al., 2004). However, in the context of the present study this seems to mirror the qualitative change between the two modes of processing but not a gradual increase of distraction. In addition, the difference between the two modes of processing also seems to include the integration of an additional processing step as mirrored in the positive component around 200 ms. In other words, the sequence of ERP components in the Distractor conditions suggests that the processing of the transients do elicit a “transient P2” component in the present study which is observable in the time window of the MMN (see Figure 2). However, for the reason that the rare stimuli in the Distractor condition seem to be processed efficiently (mirrored in the pronounced N1 amplitude) this “transient P2” is elicited very early. In contrast, in other conditions this positive component may be elicited later and overlap with the classical P3a. Importantly, from this interpretation one must conclude that this early positive component and the later positive component within the 200–400 ms time window are independent from each other. The existence of two independent fronto-central positive components following N1/MMN which might be typically intermixed in one seemingly unitary P3a would also explain why the P3a obtained in this kind of studies do not fully resemble the effects mirrored especially in the earlier ERP components (see Berti et al., 2004, 2013; Horváth et al., 2008). (An interesting idea is that P3a and “transient P2” may also fully overlap transforming the P3a into a novelty P3. But on basis of the present study this idea remains highly speculative.) However, as discussed by Berti (2008b) and Hölig and Berti (2010), the P3a time window may mirror two different aspects of attentional control in the context of distraction: One process of (automatic) disengagement or unhitching of attention from the present task (see also Polich, 2007) and another process of controlled attention, for instance, in the service of updating of task relevant information (see also Barcelo et al., 2006). It is noteworthy that the study by Berti (2008b) reports the elicitation of P3a without oddball-like presentation (with two types of equiprobable trials) demonstrating that rareness is not a necessary condition for the P3a and supporting the conclusion that the P3a does not mirror involuntary or automatic switching of attention *per se* (see Kopp et al., 2006). However, in the context of the present study it is possible that the unhitching of attention is mirrored in the early positive component (P2) as an effect of effective processing of the auditory change (as mirrored in the pronounced N1) while the controlled allocation of attention is mirrored in the (later) P3a. If this interpretation holds, one may conclude that the process of disruption of task processing by unhitching of attentional resources takes place around 200 ms (see peak of the P2 in Figure 2C). It is noteworthy that the P2 in the Distractor condition and the MMN in the Oddball condition of Experiment 1 overlap. This might be due to the fact that a deviant does not result in a strong N1 enhancement (but see the small N1 difference in Figure 2C) which again does not trigger the P2 related process. But this might be compensated by a subsequent, additional process of deviance detection based on the processing of prediction and violations from the predictions: the MMN. Importantly, if this hypothesis of two independent processes within the 200 to 400 ms time window contributing to distraction and attentional control holds, this might also explain why the N1/MMN, P3a, and RON do not form “a strongly coupled chain” as formulated by Horváth et al. (2008) and as suggested by other findings including Berti et al. (2004).

Taken together the present study demonstrates that a variety of neuro-cognitive processes is related to distraction as a pre-requisite for flexible adaptive behavior. Especially within the—comparable—early processing steps, two different mechanisms contributing to behavioral distraction were identified (see also Näätänen, 1990; Escera et al., 1998; Rinne et al., 2006; Näätänen et al., 2007; Winkler et al., 2009; Berti, 2012). This fits into the perspective of a recent review by Grimm and Escera (2012) stating that different mechanisms of auditory change detection are mirrored in the early ERP and suggesting a number of factors facilitating automatic change detection. In the present study at least two different mechanisms are observable: a transient detection mechanism mirrored by the N1 component and a deviant detection or predictive coding mechanism mirrored by the MMN component. As demonstrated here, both routes of change detection trigger processes of attentional control, presumably also including change detection processes not tapped by the present methodological approach (for instance, correlated in mid latency responses of the human ERP, see Grimm et al., 2011). However, one question remains open: Why were the distraction effects in the present study smaller (and partly absent) in the Distractor conditions? The reason for this might be due to a number of additional factors influencing the actual behavioral effect of a change including stimulus characteristics (e.g., Parmentier et al., 2011a; Berti, 2012), characteristics of the sequence of stimulation (e.g., Bendixen et al., 2007; Jankowiak and Berti, 2007; Horváth et al., 2008), or the informational content of a stimulus (Parmentier et al., 2010; Ljungberg et al., 2012; Wetzel et al., 2012; Li et al., 2013). For instance, Jankowiak and Berti (2007) demonstrated a standard facilitation effect which adds to the degree of distraction (i.e., RT difference between standard and deviant trials). In other words, the RT difference between standard and deviant trials is a mixture of potential RT facilitation effects in standard trials and potential RT prolongation in deviant trials. With regard to the auditory-visual distraction paradigm, the task irrelevant auditory stimuli serve as cues for the upcoming, task relevant visual stimulus which facilitates processing of the visual stimulus compared with a non-cued situation (see Escera et al., 1998). In addition, it has been shown that the auditory deviant only distracts the processing of the visual information if the auditory stimulation contains information that is relevant to the experimental task (see Parmentier et al., 2010; Ljungberg et al., 2012; Wetzel et al., 2012; Li et al., 2013). This demonstrates that the auditory stimulation is processed only if it is of potential benefit for the task at hand (e.g., as a cue). Interestingly, this can also lead to facilitation effects by deviants compared to standards (Parmentier et al., 2010; SanMiguel et al., 2010a,b; Wetzel et al., 2012). With regard to the present study one may conclude that the difference in distraction effects in Experiment 2 is due to a lack of additional facilitation effects by the cueing and, in this sense, the distraction effects in the Distractor condition mirror “pure” distraction. Interestingly, a facilitation or cueing effect in the Oddball condition is likely because in both experiments RT in the frequent condition is faster in the Oddball compared with the Distractor condition; Experiment 1: 420 vs. 432 ms, *t*_(11)_ = 2.243, *p* = 0.047, Cohen's *d* = 0.185; Experiment 2: 472 vs. 494 ms, *t*_(15)_ = 3.173, *p* = 0.006, Cohen's *d* = 0.334. In addition, it is also possible that in the Distraction condition of both experiments the rare auditory stimuli serve as non-informative cues because the coupling of the stimulus onset with the onset of the visual target is too lose. With this, one should expect no distraction effect of the rare stimuli at all (see Parmentier et al., 2010; Ljungberg et al., 2012; Wetzel et al., 2012; Li et al., 2013). If this interpretation holds, the behavioral distraction effect obtained in the Distractor condition of Experiment 2 can be interpreted as additional support for the notion that distraction by deviants and transients are based on two distinct routes. However, even though the ERP findings may sometimes suggest a straightforward coupling of the processing of deviant and novel stimuli to behavioral distraction, the pattern of influences and interactions between different processes of change detection and of attentional allocation resembles more gradual effects of the automatic processing of environmental sensory information on behavior. Further research may elucidate the interaction of neuronal mechanisms of sensory and attentional processing in order to provide us with a fuller picture of how the gradual and effective adaptations to a wide variety of dynamic changes in the environment is realized in humans.

### Conflict of interest statement

The author declares that the research was conducted in the absence of any commercial or financial relationships that could be construed as a potential conflict of interest.
